# Renal Effects of Glucagon-like Peptide-1 Receptor Agonists in Diabetic Kidney Disease: A Narrative Review of Mechanisms and Clinical Evidence

**DOI:** 10.3390/medicina62081509

**Published:** 2026-08-05

**Authors:** Adina Braha, Bogdan Timar, Adrian Sturza, Romulus Timar

**Affiliations:** 1 Department of Second Internal Medicine—Diabetes, Nutrition, Metabolic Diseases, and Systemic Rheumatology, “Victor Babes” University of Medicine and Pharmacy, 300041 Timisoara, Romania; braha.adina@umft.ro (A.B.); timar.romulus@umft.ro (R.T.); 2 Department of Diabetes, Nutrition and Metabolic Diseases, “Pius Brînzeu” County Emergency Clinical University Hospital, 300723 Timisoara, Romania; sturza.adrian@umft.ro; 3 Center for Molecular Research in Nephrology and Vascular Disease (MOL-NEPHRO-VASC), “Victor Babes” University of Medicine and Pharmacy, 300041 Timisoara, Romania; 4 Department of Functional Sciences-Pathophysiology, Center for Translational Research and Systems Medicine, “Victor Babes” University of Medicine and Pharmacy, 300041 Timisoara, Romania

**Keywords:** glucagon-like peptide-1 receptor agonists, diabetic kidney disease, chronic kidney disease, cardiorenal protection

## Abstract

Diabetic kidney disease (DKD) remains a major cause of advanced chronic kidney disease (CKD) and cardiovascular (CV) mortality, despite optimization of renin–angiotensin–aldosterone system (RAAS) blockade and the use of sodium–glucose cotransporter-2 inhibitors (SGLT2i). Glucagon-like peptide-1 receptor agonists (GLP-1 RAs) are cardiometabolic agents with significant efficacy on glycemic control, body weight, blood pressure (BP), lipid profile, and systemic inflammation. In experimental studies, GLP-1 RAs showed direct renal effects by modulating natriuresis, intrarenal hemodynamics, oxidative stress, endothelial dysfunction, and tubular apoptosis. Randomized clinical trials and real-life analyses have demonstrated reductions in albuminuria and slowing of glomerular filtration rate (GFR) decline. The first study with a primary renal endpoint for semaglutide confirms its nephroprotective potential. This narrative review synthesizes the renal mechanisms involved. The clinical evidence for GLP-1 RA in DKD positions this class alongside SGLT2i and non-steroidal mineralocorticoid receptor antagonists (ns-MRAs) for the management of patients with type 2 diabetes mellitus (T2D), CKD, and very high cardiorenal risk.

## 1. Introduction

Today, hundreds of millions of adults are living with diabetes mellitus (DM) worldwide [1]. Over the course of the disease, a significant proportion develop chronic kidney disease (CKD), with a major impact on survival and quality of life [2,3]. DM is currently the leading cause of CKD and end-stage renal disease, contributing decisively to cardiovascular (CV) mortality and healthcare costs (ESRD) [3,4].

CKD associated with DM (DKD) is the result of a complex interaction between hemodynamic factors (glomerular hyperfiltration, intraglomerular hypertension), metabolic mechanisms (chronic hyperglycemia, accumulation of advanced glycation end products), oxidative stress, chronic low-grade inflammation, and neurohormonal activation, particularly of the renin–angiotensin–aldosterone system (RAAS). This network of mechanisms leads, over time, to the development of albuminuria, a progressive decline in estimated glomerular filtration rate (eGFR), and ultimately to irreversible loss of renal function [5,6]. It is estimated that CKD is prevalent in approximately half of patients with DM [7], with about 33% of adults with type 1 diabetes (T1D) developing albuminuria and 25% developing reduced estimated glomerular filtration rate (eGFR) after more than 40 years of DM, and with 10–30% of them progressing to ESRD [8].

For many decades, the nephroprotective strategy in DM has been based mainly on glycemic and blood pressure control, RAAS blockade, and lifestyle modification [9]. Although these interventions remain fundamental, they have failed to halt the progression of DKD in many patients, which has highlighted an important “residual risk” for renal and CV disease (CVD). In recent years, the new approach to DKD has entered a new phase after introducing in clinical practice the sodium–glucose cotransporter type 2 inhibitors (SGLT2i) and glucagon-like peptide-1 receptor agonists (GLP-1 RA) [9], which have demonstrated the ability to modify the natural history of the disease, reducing the risk of renal progression and major CV events.

GLP-1 RAs occupy a special place in this landscape, offering a combination of effects on glucose metabolism, body weight, BP, and lipid profiles, as well as direct nephroprotective potential. Numerous clinical and experimental studies with CV endpoints have also documented the nephroprotective effects of GLP-1 RA. In addition, the existing literature sometimes addresses pathophysiological mechanisms, systemic effects, and clinical data separately, making it difficult to integrate them into a practical message for the clinician. However, information regarding their role in DKD is fragmented, scattered across several trials and secondary analyses.

The present narrative review aims to integrate the systemic and direct renal mechanisms through which GLP-1 RA may provide nephroprotection in diabetic renal disease and to critically appraise the current clinical evidence.

## 2. Methods

For the present narrative review, we conducted a structured search of the PubMed and Web of Science databases for articles published in English between January 2000 and December 2025, using combinations of keywords and Boolean operators relevant to the paper’s topic. The search strings included: “diabetic kidney disease” OR “diabetic nephropathy” OR “chronic kidney disease” AND “GLP-1 receptor agonist” OR “GLP-1 RA” AND “renal outcome” OR “kidney outcome” OR albuminuria OR “eGFR decline” AND “type 2 diabetes” AND “GLP-1” OR “incretin” AND “renal protection” OR “cardiorenal protection”. The search was last updated on 30 December 2025.

The following types of publications were considered eligible: randomized clinical trials (including CV trials with secondary or post hoc renal endpoints), randomized or nonrandomized studies with dedicated renal endpoints, large observational cohorts, and real-world analyses of the renal effects of GLP-1 RA, experimental and mechanistic studies relevant to the renal action of GLP-1 RA, guidelines, and consensus statements from major international societies.

We excluded case reports and case series; studies with very small numbers of patients without clear renal endpoints; unpublished or non-peer-reviewed materials; and purely narrative articles that did not provide additional original data. We prioritized studies with the largest number of participants, the most recent and complete analyses, and renal prespecified outcomes. Disagreements about study eligibility or prioritization were resolved by discussion and, when needed, by consultation with a third senior author. We included in the Supplementary File the PRISMA flowchart summarizing the search process [10].

Given the significant heterogeneity of reported renal outcomes, we categorized the results into three categories: hard renal outcomes (need for renal replacement therapy, end-stage renal disease, sustained and significant decline in eGFR, or death from renal causes), surrogate markers (appearance or progression of albuminuria, changes in urinary albumin/creatinine ratio, long-term slope of eGFR), and composite cardiorenal outcomes, which combine major renal and CV events. In the critical synthesis, we explicitly highlighted whether the results were from prespecified primary outcomes, secondary outcomes, or exploratory post hoc analyses.

Two authors independently screened titles and abstracts and subsequently reviewed potentially eligible full-text articles. Given the narrative nature of this review, we did not conduct a formal risk-of-bias assessment or perform quantitative meta-analyses. However, in interpreting the data, we prioritized large randomized trials, trials with dedicated renal endpoints, and evidence-based guidelines, using observational studies and real-world data to complement long-term perspectives and generalizability of results in diverse populations with DKD. The reference lists of relevant articles were hand-searched to identify additional sources for a non-systematic narrative synthesis. The study selection was guided by relevance and methodological quality.

## 3. The Pathophysiology of DKD

**Figure 1:**
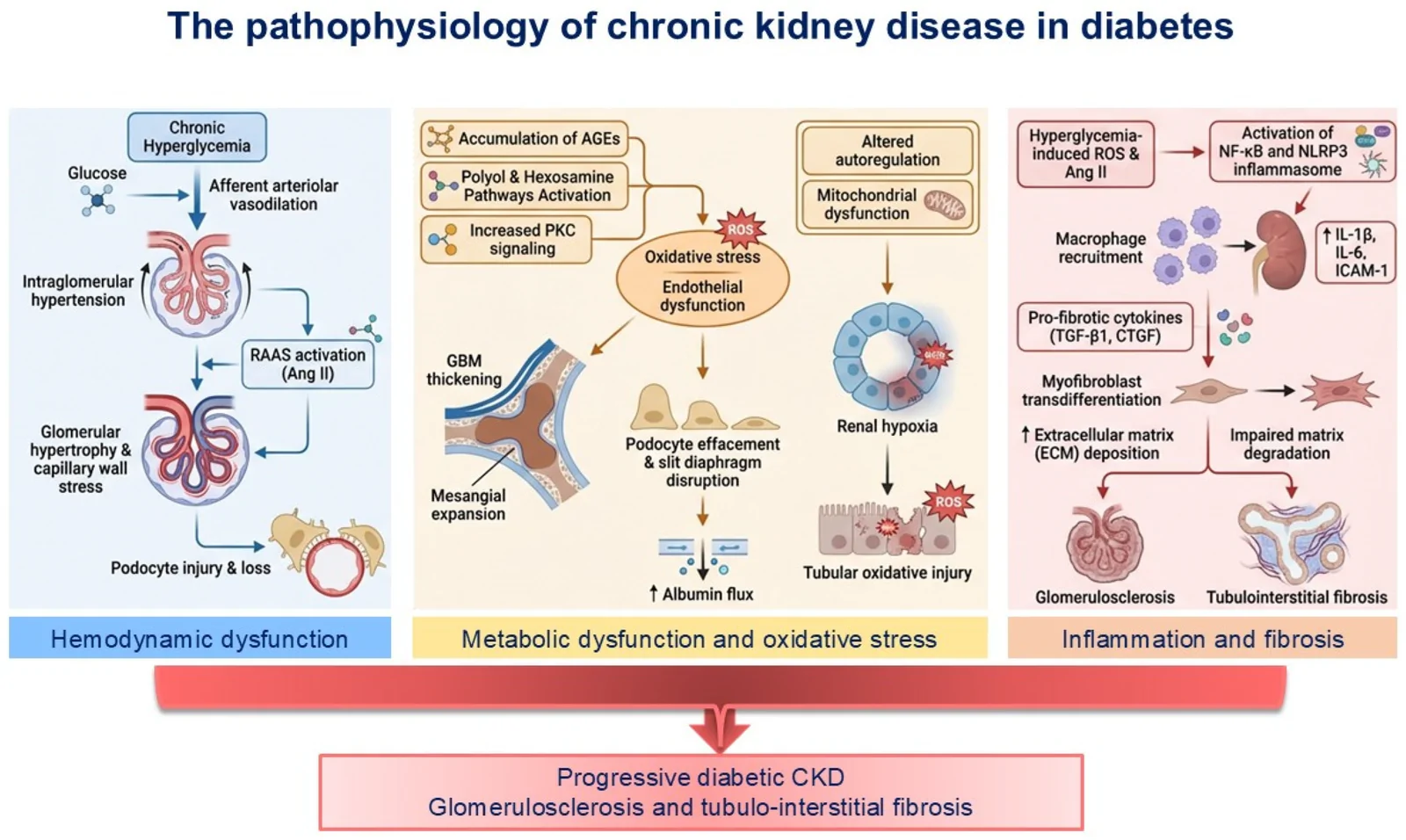
The pathophysiology of chronic kidney disease in diabetes. Abbreviations: RAAS = renin–angiotensin–aldosterone system; AGEs = advanced glycation end-products; PKC = protein kinase C; GBM = glomerular basement membrane; ROS = reactive oxygen species; NF-κβ = nuclear factor kappa-light-chain-enhancer of activated B cells; NLRP3 = NLR family pyrin domain–containing 3 inflammasome; IL-1β = interleukin-1 beta; IL-6 = interleukin-6; ICAM-1 = intercellular adhesion molecule-1; TGF-β1 = transforming growth factor-beta 1; CTGF = connective tissue growth factor; ECM = extracellular matrix; CKD = chronic kidney disease; Ang II = angiotensin II.

The pathogenesis of DKD involves complex interactions among hemodynamic, metabolic, and biochemical alterations driven by chronic hyperglycemia and other risk factors. This complex relationship becomes apparent initially as glomerular hyperfiltration and hypertrophy and, after a long period of asymptomatic years, eventually leads to glomerulosclerosis, a more rapid decline in GFR, and increased albuminuria. Figure 1 illustrates the fundamental pathways underlying DKD [11].

Chronic hyperglycemia activates multiple metabolic pathways, leading to mitochondrial dysfunction and oxidative stress in the kidney. When glucose undergoes non-enzymatic reactions with proteins, lipids, and nucleic acids, it leads to structural and functional alterations in these biomolecules, resulting in the formation of AGEs, which are accumulated in renal tissues, triggering inflammation and fibrosis via receptor-mediated mechanisms, like the receptor for advanced glycation end-products (RAGE) [12,13,14].

Chronic hyperglycemia also increases renal blood flow and GFR by vasodilating the afferent arterioles and constricting the efferent arterioles. Persistent hyperfiltration sustained by excess angiotensin II (Ang II) and RAAS activation promotes injury to glomerular capillaries, leading to mesangial hypertrophy and increased glomerular basement membrane (GBM) thickness, aggravating renal structural damage and accelerating the progression of fibrosis to glomerulosclerosis and the decline of renal function [15,16,17].

At the glomerular level, this toxic metabolic context causes endothelial dysfunction, with reduced nitric oxide bioavailability and increased expression of adhesion molecules (ICAM-1), facilitating leukocyte recruitment. Podocytes are injured, leading to podocyte effacement and slit diaphragm disruption, which increases albumin flux across the filtration barrier. In response to reactive oxygen species (ROS) and profibrotic mediators (transforming growth factor-beta 1 (TGF-β1), connective tissue growth factor (CTGF)), mesangial cells respond by expanding and increasing extracellular matrix (ECM) synthesis [18,19].

In the tubulointerstitial compartment, increased filtered albumin, ROS, and AGEs induce tubular oxidative injury and activate innate immune pathways, such as nuclear factor kappa-light-chain-enhancer of activated B cells (NF-κβ) and the NLR family pyrin domain–containing 3 inflammasome (NLRP3), leading to the release of proinflammatory cytokines (IL-1β, IL-6). This inflammation, combined with persistent Ang II signaling, promotes capillary rarefaction and renal hypoxia [20,21,22]. This oxidative stress acts synergistically with glomerular hyperfiltration, glycation, and AGE accumulation, resulting in a vicious cycle of progressive renal injury characteristic of diabetic nephropathy [23]. Angiotensin II receptor blockers and SGLT2i have been shown to reduce inflammatory signaling and fibrogenesis and slow the decline in renal function [24,25].

## 4. Disease Diagnosis and Management

CKD is diagnosed based on a persistently reduced estimated GFR ≤ 60 mL/min and micro- or macroalbuminuria for at least 3 months [26]. DKD is usually a clinical diagnosis in patients with long-standing diabetes (>10 years), regardless of type, with albuminuria and/or reduced eGFR in the absence of signs or symptoms of other primary causes of kidney damage [27].

According to the latest guidelines, the pharmacological approach in T2D focuses on individualizing targets, preventing complications, and avoiding medical inertia. For patients living with T2D who also have heart failure (HF) and CKD, selected therapies such as SGLT2i and/or GLP-1 RA are preferred based on evidence that they reduce CV and kidney disease risk [28]. In people living with DKD, the American Diabetes Association (ADA) recommends a holistic approach depending on the type of diabetes, with a risk factor reassessment every 3 to 6 months. Beyond lifestyle changes and RAAS blockade, SGLT2is are the first-line choice if eGFR > 20 mL/min and, as needed, GLP-1 RA and finerenone as an add-on to SGLT2i to mitigate CV and renal risk [9].

When we approach hypertension, the European Society of Hypertension (ESH) 2023 [29] and the American Heart Association/American College of Cardiology (AHA/ACC) 2025 [30]. Guidelines recommend reaching a therapeutic threshold of 130/80 mmHg in people with DM, with maintenance of a systolic BP < 130 mmHg in most patients. A more strict systolic BP goal of <120 mmHg should be aimed for in individuals with high CV or kidney risk [30,31]. To achieve these goals, RAAS blockade is recommended for all patients with DKD, regardless of albuminuria stage, even if BP is only mildly elevated. In addition, after optimizing treatment with angiotensin-converting enzyme (ACE) inhibitors or angiotensin II receptor blockers (ARBs), finerenone (a selective nonsteroidal mineralocorticoid receptor antagonist) is indicated in these patients with eGFR ≥ 25 mL/min/1.73 m^2^ and albuminuria A2–A3 to reduce renal progression and CV events. Calcium channel blockers (especially dihydropyridines) are common adjuncts. However, they are ideally combined with ACEI/ARB to limit the increase in albuminuria [32].

In the general population, obesity is associated with increased risk of CKD, progression to advanced stages, and CV mortality. Although obesity clearly increases the risk of developing and progressing CKD, a higher BMI was paradoxically associated with better survival in advanced CKD and in dialysis patients [33,34,35,36]. Interpreting this phenomenon requires understanding the limitations of BMI, the role of muscle mass, wasting, and survivor selection. BMI does not separate muscle mass from fat mass; part of the “protective effect” of obesity actually reflects the advantage of better muscle mass and nutritional status. Patients with lower BMI often have involuntary weight loss, inflammation, and protein-energy malnutrition, all of which are associated with increased mortality; after adjusting for prior weight loss, the “protective” association of obesity disappears in some analyses. In dialysis patients, increased extracellular volume may overestimate body weight, and elevated BMI may partly reflect hypervolemia or differences in fat distribution. Abdominal obesity and metabolic risk remain associated with CV events and mortality even in dialysis patients; in patients with advanced CKD and poor nutritional status, the target is not “weight loss at all costs”, but maintaining an optimal nutritional status while controlling cardio-metabolic risk factors [35,36].

In the population of patients with CKD and obesity, GLP-1 RA may have a dual impact: on the one hand, they reduce weight, inflammation and cardio-renal risk; on the other hand, rapid weight loss, especially if not accompanied by adequate protein intake and physical activity, may accentuate lean mass loss and precipitate a sarcopenic phenotype or “sarcopenic obesity”. Recent data suggest that GLP-1-based treatments can induce significant loss of fat mass but also of lean mass, especially at high doses and in patients with reduced muscle reserve. The treatment of obesity in patients with CKD is based on three pillars, supported by evidence of efficacy and safety: lifestyle changes tailored to the stage of CKD, pharmacotherapy (mainly GLP-1/dual GLP-1 GIP RA), and metabolic surgery in selected cases. Recent guidelines place GLP-1 RA (liraglutide, semaglutide, dulaglutide) and GLP-1/GIP co-agonists (tirzepatide) as first-line pharmacological therapies for overweight/obesity in CKD, especially when diabetes or increased CV risk coexist. CKD is already associated with an increased risk of sarcopenia through chronic inflammation, metabolic acidosis, uremia, insulin resistance, anorexia, and reduced physical activity. In patients with a “sarcopenic obesity” phenotype, elderly or frail, the renal and CV benefits and the risk of loss of muscle mass and function following obesity therapy with GLP1/GLP1-GIP agonists become questionable [33,37]. Therefore, an initial assessment of muscle mass and strength, nutritional counseling focused on adequate protein intake and maintaining or increasing muscle mass within the restrictions imposed by CKD, and a resistance exercise program adapted to the level of renal function, comorbidities, and physical capacity are recommended.

## 5. Mechanisms of Action of GLP-1 RA

The incretin axis was first reported in 1964, based on studies that concluded that oral glucose induces greater insulin secretion than intravenous glucose [38,39]. This insulin response was altered in patients with T2D [40]. The incretin system involves two hormones, glucose-dependent insulinotropic polypeptide (GIP) and GLP-1, secreted from the gastrointestinal tract in response to nutrient ingestion, which significantly enhance insulin secretion from pancreatic beta cells. Both incretins are rapidly degraded by the dipeptidyl peptidase-4 (DPP4) enzyme [40]. The GLP-1 RA mimics the native incretin GLP-1 by activating the GLP-1 receptors (GLP-1Rs) and regulating glucose metabolism. It stimulates pancreatic beta-cells to produce insulin in a glucose-dependent manner and also inhibits postprandial glucagon release [41].

Within the central nervous system, GLP-1R-expressing neurons are concentrated in hypothalamic nuclei (including the arcuate, paraventricular, dorsomedial, and supraoptic nuclei) and in brainstem structures such as the nucleus tractus solitarius (NTS) and other components of the dorsal vagal complex. GLP-1R expression has also been demonstrated in extrahypothalamic regions, including thalamic and cortical areas, as well as in circumventricular organs. Outside the brain, GLP-1R is predominantly found on pancreatic beta cells and in various peripheral tissues such as vascular smooth muscle, cardiac myocytes, adipose tissue, gastric parietal and smooth muscle cells, and myenteric plexus neurons, with particularly high expression in Brunner’s glands of the duodenum [41].

Unlike endogenous GLP-1, GLP-1 RAs have a prolonged half-life relative to the native peptide. Based on their pharmacokinetic profile, they are classified as short-acting agonists (<24 h): exenatide with immediate-release formulation (twice per day), lixisenatide (daily) with a predominantly postprandial effect, or long-acting agonists (≥24 h): liraglutide (daily); exenatide with once-weekly extended-release microsphere formulation, dulaglutide (weekly), albiglutide (weekly); semaglutide (weekly or daily orally), with a more stable action profile, predominantly affecting fasting blood glucose and HbA1c. New-generation co-agonists, such as GLP-1/GIP (tirzepatide), GLP-1/glucagon, etc., have enhanced metabolic and weight-loss effects [42,43,44,45].

### 5.1. Mechanisms of Nephroprotection of GLP-1 RA

Experimental studies suggest that GLP-1 receptors may be expressed in several renal compartments, including proximal tubular cells and preglomerular vascular smooth muscle cells, but their precise localization and abundance remain controversial and appear to depend on species, disease state, and detection methods [41,46]. In some models, GLP-1 RA exposure is associated with increased natriuresis, changes in renal hemodynamics, and improved eGFR, which have been interpreted as possible consequences of direct or indirect GLP-1R activation in tubular and vascular cells [47]. Similarly, reports of GLP-1R expression on renal macrophages and its role in modulating inflammation and fibrosis are hypothesis-generating and require further validation [37]. Therefore, renal GLP-1R expression and direct nephroprotective signaling should be viewed as plausible but not definitively established.

#### 5.1.1. The Indirect Effects of GLP-1 RA

GLP-1 has multiple physiological effects: it promotes insulin release from the pancreas in a glucose-dependent manner; inhibits glucagon release, which helps lower blood glucose levels by reducing hepatic glucose production; slows gastric emptying, contributing to increased satiety and reduced appetite; and aids weight management [48].

The beneficial systemic effects of GLP-1 RA extend beyond glycemic control. In vitro studies have shown that GLP-1 RA enhances insulin sensitivity in adipocytes by reducing macrophage production of inflammatory cytokines, including TNF-β, IL-6, and IL-1β [49]. GLP-1 RA has been shown to reduce intestinal lipid absorption by decreasing lipase activity [44], alter gut motility, and modify gut hormones, enhancing satiety and altering intestinal nutrient handling [50]. They improve lipid metabolism by reducing lipogenesis, inhibiting lipid peroxidation, and enhancing fatty acid β-oxidation, thereby reducing lipotoxicity [51]. The GLP-1 RA leads to weight loss and improved body composition by reducing visceral and ectopic fat depots of white adipose tissue [52]. Moreover, GLP-1 RAs stimulate brown adipose tissue thermogenesis and browning through hypothalamic AMPK [53].

Clinical trials have shown that GLP-1 RAs can lead to modest reductions in systolic BP, an effect attributed to increased natriuresis, inhibition of the RAAS, reduction in endothelial dysfunction, attenuation of microvascular permeability, and reduced expression of adhesion molecules [54,55]. They also exert anti-atherosclerotic effects by inhibiting oxidative stress in endothelial cells and reducing atherosclerotic lesions via AMPK- and MAPK-dependent mechanisms. GLP-1 RAs directly influence atherosclerotic plaque development and stability, reducing foam cell formation and carotid intima-media thickness and affecting the formation and progression of early-stage atherosclerosis [56]. By suppressing the proliferation and migration of these cells, GLP-1 RAs help stabilize the vascular wall and prevent excessive tissue growth after injury.

Mechanistically, these systemic effects converge on the kidney through several hemodynamic and metabolic pathways. Weight loss and the associated reduction in visceral adiposity attenuate obesity-driven glomerular hyperfiltration. In obesity-related glomerulopathy, selective afferent-arteriolar vasodilation raises intraglomerular pressure, driving podocyte stress and albuminuria. Lowering systemic blood pressure reduces the pressure transmitted to the glomerular capillary bed, limiting barotrauma to the filtration barrier and slowing the rise in albuminuria and the decline in eGFR. Improved glycaemic control lessens the formation of advanced glycation end-products and glucose-dependent tubular and mesangial injury. At the same time, improvements in the lipid profile and reductions in systemic and adipose-tissue inflammation diminish the metabolic load on the nephron. It should be emphasised, however, that these indirect effects do not appear to account for the renal benefit observed in outcome trials fully: in a secondary analysis of CV outcome data, the reduction in high-risk CKD was only partly attributable to changes in body weight, glycated haemoglobin and systolic blood pressure, implying additional, more direct renal actions of GLP-1 RA that are discussed below [57,58].

**Figure 2:**
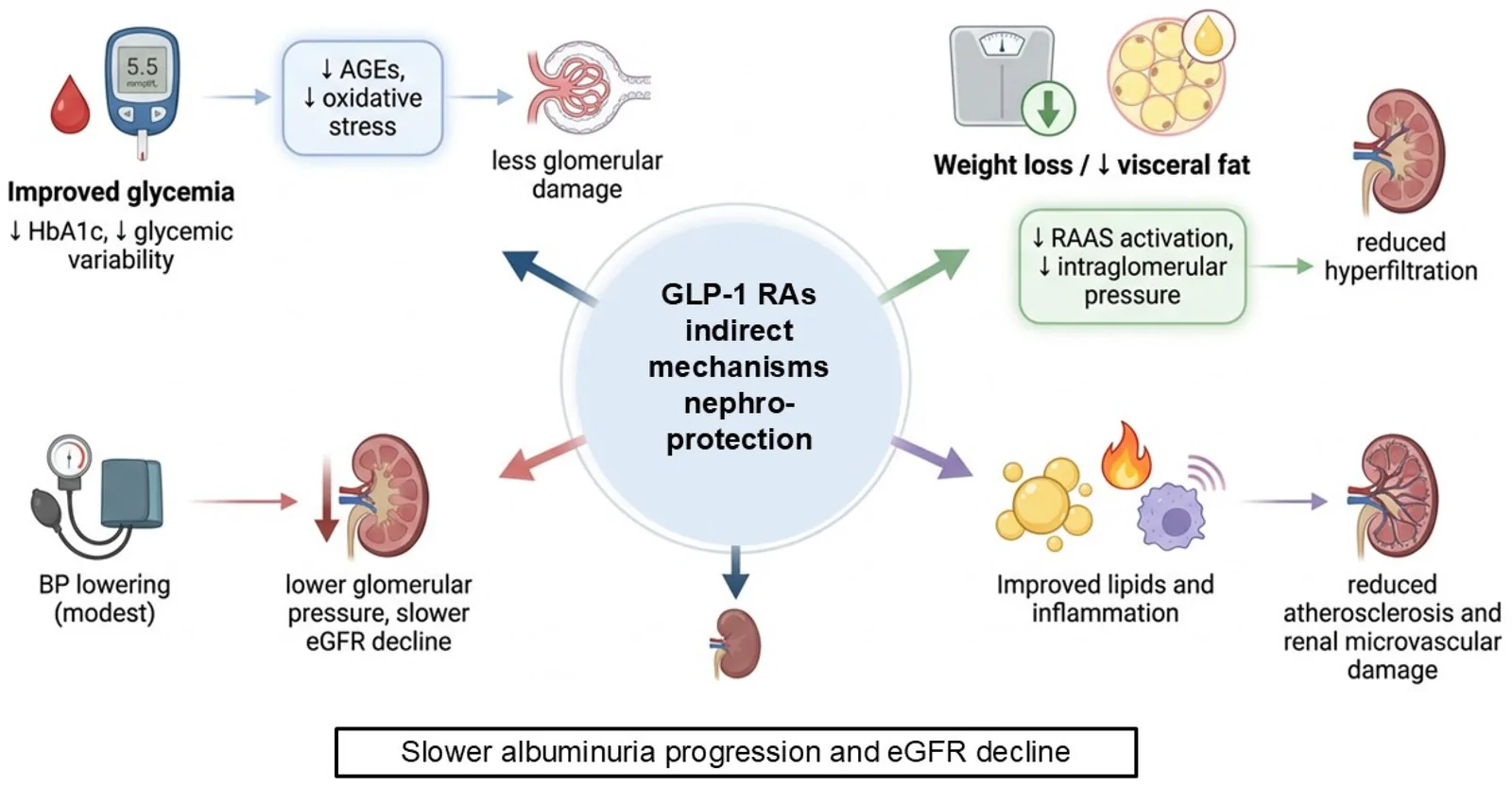
The indirect effects of GLP-1 RAs.

Figure 2 illustrates the indirect mechanisms of GLP-1 RA that slow the progression of albuminuria and eGFR decline.

#### 5.1.2. The Direct Renal Effects

Through natriuresis, the kidney maintains sodium balance and regulates BP. The sodium-hydrogen exchanger (NHE3) is a key mediator of sodium reabsorption in the proximal tubules; the more active it is, the more sodium is reabsorbed. In DKD, hyperglycemia and oxidative stress disrupt cAMP/PKA signaling, increasing NHE3 expression and activity, leading to increased sodium reabsorption, hypertension, and volume overload [59]. GLP-1 RAs modulate NHE3 by increasing cAMP production, thereby activating protein kinase A (PKA) and, as a result, inhibiting sodium reabsorption in the proximal convoluted tubule, allowing more sodium to reach the macula densa and enhancing tubuloglomerular feedback, which reduces glomerular hyperfiltration and intraglomerular pressure [60,61]. Additionally, GLP-1 RAs attenuate the effects of angiotensin II, a potent activator of NHE3, thereby further reducing sodium retention. These actions reduce glomerular hyperfiltration [62,63].

GLP-1 RAs increase natriuretic peptide activity, including atrial natriuretic peptide (ANP), resulting in increased natriuresis, which contributes to a modest decrease in BP and reduced circulating volume [54,64]. ANP is a cardiac hormone that plays a vital role in natriuresis, diuresis, and the regulation of vascular tone. ANP works by increasing glomerular filtration, blocking sodium reabsorption in the collecting tubules, and “opposing” the RAAS. In DKD, the kidney no longer responds as well to ANP because the renal endothelium is damaged and the signaling pathways are altered. In other words, even if ANP is present, its effect of eliminating salt and dilating blood vessels is reduced in DKD [65,66]. GLP-1 RAs correct the renal response to ANP by improving endothelial function, reducing oxidative stress, and normalizing signaling pathways altered in DKD. GLP-1 RAs can potentiate the effects of ANP by increasing intracellular cAMP levels [67]. In experimental studies, GLP-1Rs improved renal vascularization and oxygen supply, lowering oxygen consumption and enhancing ATP production [68].

Activation of GLP-1Rs acts on the cAMP/PKA couple, reduces proinflammatory cytokines and chemokines (TNF-α, IL-6, MCP-1), decreases macrophage infiltration into renal and vascular tissues, and inhibits the NF-κB pathway [69,70,71].

Under physiological conditions, the endothelial cells produce nitric oxide (NO), which orchestrates vasodilation and vascular tone. Also, NO inhibits leukocyte adhesion and platelet aggregation and limits the activation of the coagulation cascade. In DKD, chronic hyperglycemia, insulin resistance, and excess free radicals reduce the bioavailability of NO in the vasculature, leading to loss of endothelium-dependent vasodilation [72,73].

GLP-1 RAs increase NO production by stimulating endothelial nitric oxide synthase (eNOS) activity, which improves endothelium-dependent vasodilation and tissue perfusion. In parallel, they reduce ROS production and activate antioxidant pathways, decrease the expression of proinflammatory cytokines and leukocyte adhesion molecules at the endothelial level, and thereby reduce oxidative stress, inflammation, and the perpetuation of vascular injury [74,75].

In the context of endothelial dysfunction in DKD, GLP-1 RAs directly protect against endothelial dysfunction by improving microvascular function, reducing albuminuria, and slowing the progression of kidney disease, beyond glycemic control [76,77].

The oxidative stress that contributes to CKD results from an imbalance between the production of reactive oxygen species (ROS) and the efficacy of antioxidant systems in neutralizing them. Hyperglycemia triggers the polyol pathway, leading to the production of reactive oxygen species (ROS), the formation of advanced glycation end-products, and the activation of protein kinase C (PKC) [17,78].

Beyond glycemic control, GLP-1 RAs have direct antioxidant effects by preventing mitochondrial dysfunction and lowering NADPH oxidase levels [79,80,81].

GLP-1 RAs also boost the body’s own antioxidant defenses by activating nuclear factor erythroid 2-related factor 2 (Nrf2), which increases the levels of antioxidant enzymes like superoxide dismutase (SOD), catalase, and glutathione peroxidase [82,83,84].

Tubular injury and apoptosis are pivotal events in the progression of DKD, as metabolic and hemodynamic insults disrupt tubular epithelial cell integrity [85].

GLP-1 RAs have shown therapeutic potential in protecting against tubular injury and apoptosis in DKD. GLP-1 RAs’ protection comes through multiple mechanisms. By improving glucose control, they reduce the toxic effects of chronic hyperglycemia, including the formation of advanced glycation end products (AGEs) and activation of the polyol pathway [63,86]. In addition to their effects on glycemic control, they reduce the local inflammatory response, thereby maintaining tubular cell viability [87,88].

**Figure 3:**
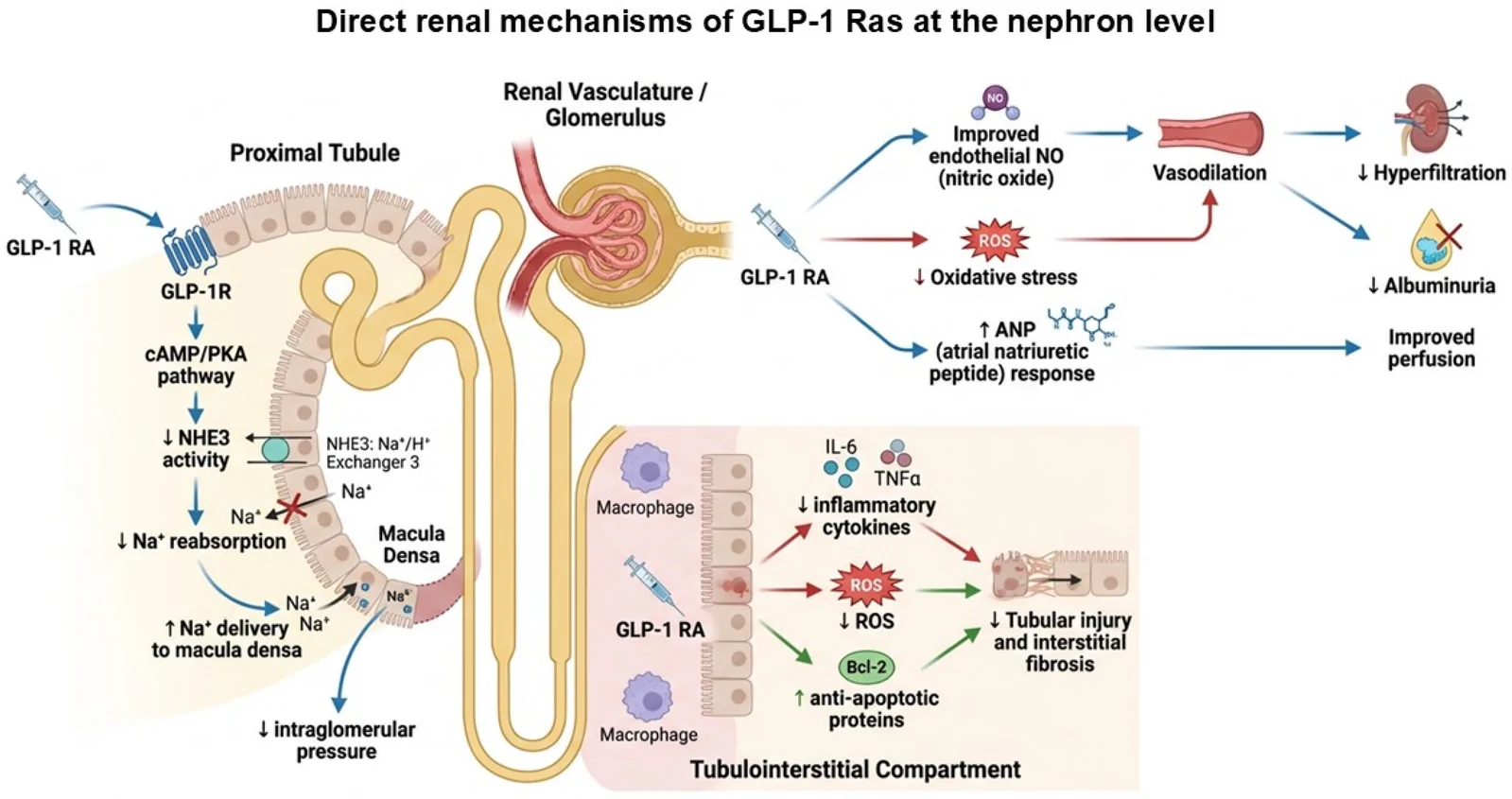
Direct renal effects of GLP-1 RAs.

GLP-1 RAs rebalance the “apoptotic balance”: they reduce the activation of pro-apoptotic molecules (such as caspases) and promote the expression of anti-apoptotic proteins (such as Bcl-2) [89,90]. By limiting epithelial cell loss and preventing the progression of acute injury to chronic fibrosis, this class helps preserve nephron structural integrity and slow the decline in renal function in DKD. The direct renal mechanisms of GLP-1 Ras are presented in Figure 3.

### 5.2. Evidence from Randomized Clinical Studies with GLP-1 RA and Renal Outcomes

GLP-1 RAs have generated a consistent body of evidence regarding renal protection, both from randomized CV outcome trials, meta-analyses [91], and from dedicated renal studies and real-life cohorts. The GLP-1 RA trials (ELIXA [92], LEADER [93], SUSTAIN-6 [94], EXSCEL [95], REWIND [96], AMPLITUDE-O [97], PIONEER 6 [98], SCALE [99]) primarily monitored CV events but also measured secondary renal parameters (new-onset or persistent macroalbuminuria, eGFR decline, need for dialysis), and renal objectives were secondary endpoints. Overall, these studies showed a consistent reduction in albuminuria and a modest slowing of eGFR decline. When the results of several large trials (e.g., LEADER and SUSTAIN-6) are combined, a clear pattern emerges: GLP-1 RAs protect the kidney primarily by reducing albuminuria and decreasing the number of major CV events. On strict renal outcomes—such as the need for dialysis or the development of end-stage renal disease—the benefit of GLP-1 RA is present, especially by decreasing albuminuria and reducing CV events, but less pronounced than that observed with SGLT2i [100].

Small studies focused on the kidneys (LIRA-RENAL [101], HARMONY 8 [102], AWARD-7 [103], PIONEER 5 [104]) showed that in patients with CKD who received GLP-1 RA, eGFR stabilizes or decreases more slowly, albuminuria (UACR) decreases, and the drugs are well tolerated, including at lower eGFR.

FLOW [105] represents the main evidence from a randomized, controlled trial (RCT) with a primary renal endpoint on the effects of semaglutide on the evolution of CKD in patients with T2D. FLOW included patients with T2D and moderate-to-advanced CKD, characterized by low eGFR and significant albuminuria, already on standard treatment (RAAS blockade and, frequently, SGLT2i), which gives the study population a high renal and CV risk. The primary endpoint was a pre-specified renal composite, which included a sustained and relevant decline in eGFR, the onset of end-stage renal failure (requiring renal replacement therapy), and certain forms of renal-related death. Strengths of the study include the large sample size, long follow-up, use of a well-defined primary renal endpoint, and integration of modern standard treatments for CKD associated with T2D into the protocol. However, the fact that the study was designed from the outset with a renal primary endpoint differentiates FLOW from most CVOTs with GLP-1 RA, in which renal was predominantly a secondary or exploratory endpoint and in which benefit was largely driven by reduction in albuminuria. However, several important limitations should be noted in interpreting the results: exclusion of patients with very low eGFR reduces generalizability to the most advanced stages of CKD, use of a composite endpoint may dilute information on each individual component, and selection of a high-risk population makes extrapolation to patients with milder CKD or no albuminuria warrant caution. Overall, FLOW supports the hypothesis of a nephroprotective effect of semaglutide. However, a nuanced interpretation is necessary, integrating both the structure of the endpoint and the profile of the included population.

When interpreting these data, the strength of the underlying evidence should be carefully weighed, as the renal findings differ across endpoint type and analytic status. Most cardiovascular outcome trials assessed kidney effects through composite outcomes dominated by changes in albuminuria, a surrogate marker, and these analyses were generally prespecified secondary or exploratory (post hoc) endpoints rather than primary outcomes powered for renal events. Hard renal endpoints, such as sustained decline in eGFR, progression to kidney failure, or renal death, were rarely the primary focus, with the notable exception of the FLOW trial of semaglutide, the first dedicated kidney-outcome trial of a GLP-1 RA, which reported a reduction in major kidney events. A subsequent meta-analysis of randomized controlled trials in T2D (involving more than 67,000 participants) reported an approximately 18% relative reduction in a composite kidney outcome and a 16% reduction in kidney failure. However, the contribution of individual molecules and the consistency of effects across albuminuria categories and baseline renal function remained heterogeneous [106]. Differences in trial populations, background therapy (including variable use of SGLT2i and RAAS blockade), follow-up duration, and the specific agent and dose studied all limit direct comparisons between trials. Accordingly, the apparent class-level nephroprotection should be regarded as a plausible but not yet uniformly established effect, and findings derived largely from surrogate endpoints and secondary analyses warrant cautious interpretation pending further dedicated kidney-outcome trials [57,58].

Table 1 summarizes the main findings of the randomized trials of GLP-1 RAs and the renal outcomes in patients with T2D.

In routine clinical practice, observational data from registries and large databases suggest that GLP-1 RAs are associated with a slower decline in eGFR and a reduced incidence of severe renal events, without consistent evidence of nephrotoxicity. In comparative analyses with SGLT2i, GLP-1 RA maintain a favorable nephroprotective profile. However, the magnitude of renal benefit appears generally inferior to that seen with SGLT2i, which remains the mainstay of renal protection. Table 2 summarizes evidence from real-world studies on kidney outcomes with GLP-1 RA compared with other glucose-lowering therapies.

### 5.3. Guideline Positioning of GLP-1 RAs in Diabetic CKD

GLP-1 RA could exert a modest renal benefit alongside CV protection, justifying its role as a second-line drug in patients with T2D and CKD who are not achieving glycemic control with SGLT-2i.

The KDIGO Diabetes in CKD Guidelines recommend SGLT2i as baseline therapy in patients with T2D and CKD (eGFR 20–25 mL/min/1.73 m^2^), in addition to standard RAAS blocker therapy. GLP-1 RAs are recommended as add-on second-line therapy in patients with T2D and CKD who do not achieve glycemic goals with metformin and SGLT2i, or have contraindications/intolerance to SGLT2i, given their documented CV risk-reducing effects and moderate renal benefits (especially on albuminuria and slowing of eGFR decline). The 2024 KDIGO Guidelines for the Evaluation and Management of CKD mention GLP-1 RA as part of the cardiorenal arsenal, emphasizing their role in patients with obesity, CVD, and diabetes, as well as in patients with mild to advanced CKD, when integrated with SGLT2i and RAAS blockade [26].

The ADA lists GLP-1 RA with demonstrated CV benefits (liraglutide, semaglutide, dulaglutide) as first-line options for reducing CV risk in patients with T2D and atherosclerotic CVD, regardless of HbA1c or metformin use. For patients with CKD, the ADA recommends SGLT2i with evidence of renal protection as a first-line option when eGFR allows; GLP-1 RA with CV benefits and “kidney-friendly” properties as an add-on if glycemic control remains suboptimal or if SGLT2i cannot be used. Thus, GLP-1 RAs are considered second-line therapy with renal and CV benefits and are justified for use in T2D + CKD not glycemic-controlled on SGLT2i [9].

The ADA–EASD joint report [116] emphasizes a risk-centered approach:

- In T2D with atherosclerotic CVD, GLP-1 RA with CV benefits or SGLT2i are recommended early, regardless of HbA1c;
- In T2D with CKD (especially with albuminuria), SGLT2i are preferred for renal protection, but GLP-1 RAs are additionally recommended for glycemic control, weight loss, and additional CV risk reduction. The consensus concludes that GLP-1 RA “could exert a modest renal benefit alongside CV protection”, supporting their place as second-line in T2D and CKD after SGLT2i, especially when weight loss and CV risk optimization are needed.

The ESC 2023 Guidelines for Cardiovascular Disease in Patients with Diabetes place GLP-1 RA and SGLT2i at the heart of CV risk reduction therapy. In patients with T2D and atherosclerotic CVD or very high risk, GLP-1 RAs with demonstrated CV benefits are recommended for secondary prevention, regardless of HbA1c or background therapy [117].

In the presence of CKD, the ESC recommends the combination of SGLT2i and GLP-1 RA to maximize cardio-renal protection: SGLT2i as first line to reduce CKD progression, GLP-1 RA to reduce CV events, and for their complementary nephroprotective effect [117].

Therefore, in clinical practice, we can initiate GLP-1 RA primarily for renal protection:

− In patients with T2D and CKD (eGFR 20–60 mL/min/1.73 m^2^ or albuminuria ≥ 30 mg/g) in whom CV/renal risk is high, even if the HbA1c target is approximately achieved;
− In patients with eGFR < 30 mL/min/1.73 m^2^, in whom the glycemic effect of SGLT2i is limited, but the risk of renal progression and CV events remains elevated;
− In patients with CKD and significant obesity, hypertension and persistent albuminuria under RAASi and SGLT2i, in whom weight loss and albuminuria reduction may contribute to renal protection, with careful monitoring of muscle mass in the elderly.

If the primary therapeutic target is glycemic control and CV risk reduction, and renal benefit is secondary, we can initiate GLP-1 RA.

− In patients with T2D without obvious CKD, but with documented atherosclerotic CV disease or very high CV risk, to reduce MACE and mortality, with added renal protection;
− In patients with HbA1c well above target, requiring intensification, in whom GLP-1 RA is preferred over insulin due to the lower risk of hypoglycemia and favorable effects on weight and CV.

### 5.4. Side Effects

The side effects of GLP-1 RA are presented in Table 3, including recommendations for preventing or reducing these side effects.

(a) Nausea, vomiting, diarrhea, or other gastrointestinal effects

Nausea, vomiting, early satiety, bloating, abdominal pain, and diarrhea/constipation are the most typical reactions. They occur mainly at initiation and after dose increases, are generally dose-dependent and transient, and are generally dose-dependent and transient. These symptoms may lead to decreased fluid and food intake; in patients with CKD, especially the elderly or those on multiple medications (diuretics, RAAS inhibitors), this may precipitate dehydration and acute functional renal failure [118].

Delayed gastric emptying may worsen or mimic diabetic gastroparesis (fullness, postprandial nausea, eructation). In patients with established gastroparesis, GLP-1 RA should be used with great caution or avoided to avoid worsening symptoms. In the context of CKD, where polypharmacy is common, GLP-1 RA-induced delay in gastric emptying may alter the rate and extent of absorption of co-administered oral drugs. This is particularly relevant for agents with a narrow therapeutic window or time-dependent absorption. Phosphate binders and bile-acid sequestrant resins are not themselves systemically absorbed; rather, they act within the intestinal lumen to bind dietary phosphate, bile acids, and other co-ingested drugs. Because their efficacy and potential to chelate other medications depend on luminal contact with food and concomitant drugs, slowed gastric emptying may shift the timing of these interactions, so the dosing schedules of interacting oral agents may need to be adjusted accordingly [119].

(b) Risk for acute biliary disease

Cases of acute pancreatitis have been reported in patients treated with GLP-1 RA; the causal relationship is not completely clear, but the class is considered with caution in patients with a history of pancreatitis [120]. The drug should be discontinued if suggestive abdominal pain (intense, persistent, posteriorly radiating pain) occurs. GLP-1 RAs increase the risk of gallstones and cholecystitis, probably mediated by rapid weight loss and changes in gallbladder motility. Patients with a history of cholecystopathy should be monitored clinically, and the occurrence of biliary colic requires therapeutic reevaluation [121].

(c) Increased risk for medullary thyroid cancer

Studies in rodents have shown a dose- and duration-dependent increase in C-cell tumors (medullary thyroid carcinoma), which led to the introduction of boxed warnings and a class contraindication in patients with a personal or family history of medullary thyroid carcinoma or MEN2 syndrome. For the rest of the population, current guidelines consider that the cardiorenal metabolic benefits outweigh the potential risks.

(d) Ophthalmological side effects

In some large studies (particularly with semaglutide and, less clearly, with liraglutide), an increase in complications of diabetic retinopathy (progression to proliferative retinopathy, vitreous hemorrhages, increased need for laser photocoagulation or intravitreal injections) has been observed in patients who already had retinopathy and in whom glycemias were lowered very rapidly. In recent years, reports of non-arteritic anterior ischemic optic neuropathy (NAION) have emerged in patients treated with semaglutide. NAION manifests with sudden, painless, usually unilateral loss of vision, visual field defect (often altitudinal), and papilledema on fundus examination.

(e) Cardiovascular and hemodynamic effects

The class as a whole tends to slightly reduce BP (through weight loss, natriuresis, and vascular effects), which is generally beneficial. In patients with CKD, especially those with borderline BP or intensive antihypertensive treatment, the association of the hypotensive effect with volume depletion (nausea/vomiting) may lead to symptomatic hypotension and renal hypoperfusion. A slight increase in heart rate (usually 2–4 beats/min) may be observed, often without major clinical impact. However, it is relevant in patients with tachyarrhythmias or advanced HF [122].

(f) Specific considerations for CKD

Predominantly renally excreted and short-acting agents (exenatide, lixisenatide) have a higher risk of accumulation and adverse reactions at eGFR < 30 mL/min/1.73 m^2^; therefore, they are usually avoided in advanced CKD and dialysis. Long-acting analogs (liraglutide, dulaglutide, semaglutide) do not require dose adjustment based on eGFR. They can be used until advanced stages, with monitoring of volume status and renal function, especially in the first weeks or during intercurrent episodes. At any level of eGFR, the key is prophylaxis of hypovolemia (education on fluid intake, temporary interruption in case of vomiting/febrile diarrhea) and monitoring of creatinine and potassium when the clinical picture suggests renal damage [57].

Semaglutide and liraglutide are not recommended in patients with eGFR < 15 mL/min/1.73 m^2^. Dulaglutide and Lixisenatide are not recommended in patients with eGFR < 30 mL/min/1.73 m^2^. Extended-release exenatide (LAR): not recommended in eGFR < 30 mL/min/1.73 m^2^; special caution is required in the range of 30–50 mL/min/1.73 m^2^, with close monitoring of renal function and hydration status [123].

In practice, the class toxicity of GLP-1 RA is dominated by gastrointestinal effects and their hemodynamic consequences, not by direct renal injury. In patients with CKD, these drugs remain useful and relatively safe, provided they are introduced gradually, carefully monitored, and integrated into a plan that considers circulating volume, concomitant medications, and ocular, pancreatic, and biliary status.

### 5.5. Combination Therapy: GLP-1 RA, SGLT2i and RAASi

In recent years, the DKD approach in patients with T2D has evolved from a single key class to a pillared combination therapy model, in which RAASi, SGLT2i, GLP-1 RA, and nsMRA (finerenone) act complementarily on cardiorenal risk. GLP-1 RA and SGLT2i act on different but convergent pathways: the former corrects obesity, insulin resistance, inflammation, and atherosclerosis, and the latter modulates glomerular hemodynamics, induces natriuresis, and reduces hyperfiltration. Combined, they provide a double “shield” for the diabetic kidney: they reduce albuminuria and slow the decline in eGFR through complementary mechanisms—metabolic-anti-inflammatory (GLP-1 RA) and tubuloglomerular-hemodynamic (SGLT2i)—with consistent benefits also on major CV events [88,124]. In the AMPLITUDE-O study, a significant proportion of patients were already receiving SGLT2i. The additional effects of GLP-1 RA on renal and CV composites suggest that these two classes do not overlap but are potentiated [125].

Recent KDIGO and ADA guidelines recommend the maximum tolerated use of RAAS blockers, early initiation of SGLT2i at eGFR ≥ 20 mL/min/1.73 m^2^ and significant albuminuria, the addition of a long-acting GLP-1 RA for patients in whom glycemic or CV risk targets are not reached, and in case of persistent albuminuria (UACR ≥ 30 mg/g) and eGFR ≥ 25 mL/min/1.73 m^2^, the introduction of an nsMRA such as finerenone [9,26]. Data from FIDELITY [126] and recent meta-analyses [91] suggest that combining SGLT2i with GLP-1 RA further reduces MACE, mortality, hospitalizations for heart failure, and serious renal events compared with monotherapy. The addition of finerenone on top of RAASi + SGLT2i (and sometimes + GLP-1 RA) provides additional reductions in albuminuria and DKD progression, with a manageable risk of hyperkalemia. These observations support the concept of cardiorenal triple therapy (RAASi + SGLT2i + GLP-1 RA, to which nsMRA is added in the presence of persistent albuminuria) for patients with T2D, CKD, and very high risk.

### 5.6. Dual Agonists—GLP-1 and GIP (Tirzepatide)—And Triple Agonists—GLP-1, GIP, and Glucagon (Retatrutide)

Into this landscape, the new generation of dual incretin agonists, such as tirzepatide (GLP-1/GIP), is introduced, further amplifying the already known systemic effects of GLP-1 RA. The SURPASS program [127,128,129,130,131] demonstrated reductions in HbA1c superior to both basal insulin and a “pure” GLP-1 RA, associated with impressive weight reductions (often >15% of body weight), marked improvement in insulin resistance, and further reductions in BP and atherogenic dyslipidemia. These indirect systemic effects—less visceral fat mass, improved lipid profile, and lower BP—are essential in a context of diabetic CKD, where obesity, hypertension, and atherosclerosis inexorably accelerate the loss of kidney function.

Dual agonists also exert direct effects on tissues: they reduce inflammation and oxidative stress, attenuate RAAS activation and profibrotic signaling, improve endothelial function, and favorably affect microvascular hemodynamics, including at the renal level. The result is reduced albuminuria and slowed eGFR decline [57].

Retatrutide, a peptide analog that simultaneously activates GLP-1, GIP, and glucagon receptors, leading to significant weight loss surpassing that of tirzepatide, is currently being studied in an ongoing phase 2 trial (NCT05936151) with the primary objective of modifying GFR in patients with overweight/obesity and CKD with or without T2D. However, so far, we can only anticipate that it improves kidney function through its marked beneficial effects on weight, glycemic control, lipid profile, and other cardiovascular risk factors. There is still no firm evidence that retatrutide has a “direct” nephroprotective effect similar to that of SGLT2 inhibitors or GLP-1 RAs, which have proven efficacy in clinical trials with renal endpoints [132].

In the context of diabetic nephropathy, this therapeutic triad—SGLT2i as a renal hemodynamic “background”, GLP-1 RA as a cardio-metabolic “modulator”, and possibly a dual GLP-1/GIP agonist as an incretin arm enhancement—outlines an integrated strategy to slow the progression of renal disease [124]. However, the direct nephroprotective evidence for these newer agents remains preliminary or unavailable.

### 5.7. Future Directions

An important direction of development is represented by oral nonpeptide GLP-1 agonists, such as danuglipron, lotiglipron, or orforglipron [133], small-molecule GLP-1 RAs administered once or twice daily, without restrictions on food or liquids. Danuglipron and lotiglipron have shown moderate benefits for glycemic control and weight in studies conducted to date. However, their development has been slowed by gastrointestinal or hepatic side effects [134]. Orforglipron is another small nonpeptide molecule that appears to have partial agonism with biased agonism, favoring G-protein signaling and cAMP accumulation over β-arrestin recruitment. It binds to the same GLP-1R site as the endogenous hormone, stimulating glucose-dependent insulin secretion and inhibiting glucagon secretion. In phase 3 ATTAIN-1 and ATTAIN-2 trials with individuals with obesity, respectively obesity and T2D, orforglipron demonstrated significant weight loss (≈10–12% on average) and improvements in CV risk markers. There is also a phase 3 program with a CV/renal outcome study (NCT07241390) [135] evaluating a composite endpoint including MACE, hospitalization for HF, and severe renal events (≥40% eGFR decrease, end-stage kidney disease, death). However, there is no consolidated evidence yet regarding nephroprotection [136,137,138,139].

Another future therapeutic target would be Piezo-1, a mechanosensory ion channel permeable mainly to calcium and involved in transducing mechanical stimuli into electrochemical and cellular inflammatory signals. It seems to be involved in the progression of DKD through mechanisms of mechanotransduction, inflammation, and endothelial damage, but its relationship with GLP-1 RA therapies remains indirect for now: GLP-1 RA acts at the GLP-1 receptor, while Piezo-1 modulates the production of endogenous GLP-1. However, there is currently no clinical evidence to support its practical role in the use of GLP-1 RA in patients with DKD [140,141].

## 6. Conclusions

GLP-1 RAs represent a cardio-metabolic therapeutic class with consistent but moderate nephroprotective effects. They are considered second-line in the management of CKD after SGLT2i. Numerous major CV trials and kidney-specific studies, culminating in the FLOW study, suggest that GLP-1 RA can reduce macroalbuminuria and may slow the progression of renal function decline. The KDIGO, ADA/EASD, and ESC guidelines converge in recommending an integrated cardio-renal-metabolic strategy that involves GLP-1 RA as second-line therapy in T2D with CKD and/or ASCVD, in combination with SGLT2i and RAAS blockade.

GLP-1 RA acts on multiple pathogenic pathways in DKD (hyperfiltration, inflammation, oxidative stress, fibrosis, endothelial dysfunction), combining direct renal effects (natriuresis, modulation of NHE3, improvement of ANP response, optimization of cAMP/PKA signaling) with favorable systemic effects (weight, BP, lipids, inflammation).

This multimodal approach may help explain why the renal benefit, although less pronounced than that of SGLT2i, appears relatively consistent across trials, especially in slowing albuminuria progression and slowing the eGFR slope, alongside a parallel reduction in major CV events.

In CKD, their use requires a pragmatic approach: slow titration, monitoring of volume status and renal function, and precautions in elderly, frail patients with sarcopenia, gastroparesis, or increased risk of biliary complications and retinopathy.

Dual and triple agonists, as well as the new oral nonpeptide GLP-1 RA, offer potential additional cardio-renal protection. However, evidence of direct nephroprotection is still preliminary. More research is needed to understand how they function at the molecular level and to ensure they are used correctly in clinical practice.

## Figures and Tables

**Table 1:** Randomized trials of GLP-1 RAs and renal outcomes in T2D.

Trial (Drug)	Population	Control Group n	GLP-1 RA Group n	Renal Outcome	% DM	Follow-Up (Months)	Baseline eGFR (mL/min/1.73 m^2^)	Baseline Albuminuria	Kidney Composite Outcome RR (CI)	Loss of Kidney Function RR (CI)	eGFR Change MD (mL/min/1.73 m^2^/Year)	UACr Change SMD	Macroalbuminuria RR (CI)
ELIXA [92] (lixisenatide)	Adults with T2D with high CV risk	3034	3034	Exploratory; albuminuria-driven (UACR/macroalbuminuria)	100	24.9	76.0	10.3 mg/g	0.91 (0.76–1.08)	0.85 (0.55–1.34)	0.36	−0.18	0.84 (0.69–1.03)
LEADER [93] (liraglutide)	Adults with T2D with high CV risk	4672	4668	Prespecified secondary renal composite; mainly macroalbuminuria-driven	100	46.0	80.0	macroalbuminuria 10.4%	0.80 (0.67–0.92)	0.90 (0.67–1.20)	0.13	−0.03	0.75 (0.61–0.92)
SUSTAIN-6 [94] (semaglutide ow)	Adults with T2D with high CV risk	1649	1648	Prespecified secondary nephropathy composite; largely albuminuria-driven	100	25.1	76.1	38.6 mg/g	0.62 (0.46–0.85)	1.29 (0.64–2.58)	0.60	−0.23	0.54 (0.38–0.78)
PIONEER-6 [98] (daily oral semaglutide)	Adults with T2D with high CV risk	1592	1591	Secondary/exploratory renal safety endpoints	100	15.9	75	N/A	-	0.75 (0.36–1.58)	0.55	-	-
EXSCEL [95] (exenatide QW)	Adults with T2D	7396	7356	Post hoc/exploratory renal analyses (eGFR slope/composite)	100	38.4	76.3	N/A	0.93 (0.78–1.10)	0.90 (0.76–1.07)	0.07	-	0.88 (0.71–1.08)
REWIND [96] (dulaglutide)	Adults with T2D	4952	4949	Prespecified secondary renal composite	100	72.0	75.0	1.84 mg/g	0.87 (95% CI 0.80–0.95)	0.91 (0.80–1.02)	0.08	−0.06	0.79 (0.70–0.88)
AMPLITUDE-O [97] (efpeglenatide)	Adults with T2D with high CV risk or CKD	1359	2717	Prespecified secondary renal composite (eGFR + macroalbuminuria)	100	21.7	72.4	28.3 mg/g	0.71 (0.61–0.82)	-	-	−0.18 (in pooled data)	0.71 (0.61–0.83)
SCALE [99] (Liraglutide 3,0 mg)	Adults with obesity or overweight (without diabetes)	1244	2487	Exploratory renal safety (albuminuria/eGFR)	0	13	90	N/A	-	-	-	-	-
LIRA-RENAL [101] (Liraglutide)	Adults with T2D (moderate CKD)	137	140	Secondary/exploratory renal endpoints (UACR/eGFR)	100	6	45.4	62.6 mg/g	1.00	-	0.0	−0.08	-
HARMONY 8 [102] (albiglutide)	Adults with T2D and CV disease	4732	4731	Secondary/exploratory renal endpoints	100	19.2	79.0	N/A	0.96	-	1.24	-	-
AWARD-7 [103] (dulaglutide)	Adults with T2D with moderate to severe CKD	194	382	Primary renal (eGFR slope); secondary composite/albuminuria	100	12	38.3	214.2 mg/g	0.63 (0.36–1.09)	0.76 (0.28–2.11)	1.60	−0.04	-
PIONEER 5 [104] (oral semaglutide)	Adults with T2D and moderate renal impairment (eGFR 30–59)	132	163	Secondary renal safety endpoints (eGFR/albuminuria)	100	6	45	-	-	-	Very small difference	-	-
FLOW [105] (semaglutide ow 1 mg)	Adults with T2D and CKD	1766	1767	Primary renal composite endpoint	100	40.8	47.0	567.6 mg/g	0.84 (0.71–0.99)	0.77 (0.64–0.94)	1.16	−0.16	-
ChiCTR-1701082 (Exenatide 2 mg) [107]	Adults with T2D and high BMI	80	79	Exploratory albuminuria/eGFR endpoints	100	5.5	N/A	80 mg/L	-	-	-	−0.41	-
GRADE (liraglutide) [101]	Adults with T2D	3785	1275	secondary and exploratory	100	60	94.9	6.4 mg/g	1.04 (0.87–1.23)	1.06 (0.61–1.84)	0.06	-	-
SURPASS4 (tirzepatide ow) [108]	Adults with T2D, high BMI and high CV risk	995	1000	secondary and exploratory	100	12	81.3	15.0 mg/g	0.61 (0.45–0.82)	0.72 (0.43–1.19)	2.20	−0.15	0.41 (0.26–0.65)

Abbreviations: MD = Mean Difference; SMD = Standardized Mean Difference; RR, risk ratio; CI, confidence interval; GLP-1 RA, glucagon-like peptide-1 receptor agonist; T2D, type 2 diabetes; CV, cardiovascular; CKD, chronic kidney disease; eGFR, estimated glomerular filtration rate; UACR, urine albumin-to-creatinine ratio; ow, once a week.

**Table 2:** Evidence from real-world studies of GLP-1 RAs on kidney outcomes compared with other glucose-lowering therapies.

Study	Population	Control (n)	GLP-1 RA Group (n)	Baseline eGFR mL/min/1.73 m^2^)	Baseline Albuminuria	Renal Outcome	Follow-Up (Months)	Results	eGFR Change MD (mL/min/1.73 m^2^/Year)
Boye 2018 (dulaglutide) [109]	retrospective real-world cohort; adults with T2D	1183 (glargine)	1222	0.9% had baseline eGFR < 30 and ≥15; 0.1% had <15 mL/min/1.73 m^2^	N/A	exploratory change in eGFR	12	3.3% vs. 4.1% 30% eGFR reduction (*p* < 0.0001 for dulaglutid)	+0.5
Boye 2018 (dulaglutide) [110]	retrospective EHR analysis, United States; T2D	37,074 (other drugs)	3225	variable	N/A	incident renal impairment (e.g., eGFR < 30 mL/min/1.73 m^2^)	12	smaller likelihood of having a ≥30% reduction in eGFR (2.19 vs. 3.14%; *p* < 0.0001 for dulaglutide)	+0.23
Franzén 2020 [111]	nationwide Scandinavian cohort; T2D on GLP-1 RA or other therapies	722 (DPP-4 inhibitors)	570 (liraglutide 92.5%, exenatide 6.2%, lixisenatide 0.7%, and dulaglutide 0.6%)	N/A	N/A	serious renal events (acute kidney injury requiring hospitalization, initiation of dialysis, kidney transplantation, renal death) HR 0.76 (0.68–0.85)	36	renal replacement therapy: HR 0.73 (95% CI 0.62–0.87). Hospitalization for renal events: HR 0.73 (95% CI 0.65–0.83). Death from renal causes: HR 0.72 (95% CI 0.48–1.10)	N/A
Lugner 2021 [112]	nationwide observational study; T2D	12,097 (SGLT2i)	9684	91.32	microalbuminuria 20.7%; Macroalbuminuria 4.5%	severe renal disease and transplantation, diabetic nephropathy; renal outcomes did not differ	19 (GLP-1 RA) and 13) SGLT2i)	SGLT2i showed a larger relative risk reduction for cardiorenal outcomes than GLP-1 RA (HR for composite cardiorenal outcome with SGLT2i vs. GLP-1 RA ≈ 0.85; 95% CI < 1)	HR 0.89 (0.77–1.04)
Xie 2020 [113]	target-trial emulation using US healthcare databases; T2D	other drugs (SGLT2i, DPP-4 inhibitors, SU)	GLP-1 RA	N/A	N/A	composite kidney outcome (e.g., sustained eGFR decline, kidney failure, or kidney-related death)	14	compared with SU: SGLT2i HR ≈ 0.58; GLP-1 RA HR ≈ 0.79; DPP-4i HR ≈ 0.90 for kidney outcomes (all vs. sulfonylureas; HR < 1 favour active drug)	N/A
Ueda 2022 [114]	Scandinavian cohort study; T2D initiating SGLT2i or GLP-1 RA	SGLT2i	GLP-1 RA	N/A	N/A	renal replacement therapy (HR 0.74 [95% CI 0.56–0.97]) and hospitalization for renal events (HR 0.75 [95% CI 0.65–0.88]) favoring SGLT2i	median 18 months for SGLT2i and 26 months for GLP-1 RA	SGLT2i is associated with a lower risk of kidney outcomes than GLP-1 RA (HR for kidney events with SGLT2i vs. GLP-1 RA < 1, statistically significant). At the same time, both classes improved outcomes vs. background therapy	N/A
Baviera 2022 [115]	Italian cohort; T2D in routine practice	39,471 (SGLT2i)	21,960	N/A	N/A	renal disease as primary diagnosis (patients with advanced renal disease)	from the first prescription of GLP-1 RA or SGLT-2i until the occurrence of one of the outcomes	overall effectiveness and safety are broadly comparable; a small advantage of SGLT2i on kidney outcomes (HR for renal events slightly <1 in favour of SGLT2i), but overlapping confidence intervals for some endpoints	N/A

Abbreviations: GLP-1 RA: glucagon-like peptide-1 receptor agonist; SGLT2i: sodium–glucose cotransporter 2 inhibitor; DPP-4i: dipeptidyl peptidase-4 inhibitor; SU: sulfonylureas; eGFR: estimated glomerular filtration rate; EHR: electronic health record; HR: hazard ratio; CI: confidence interval; MD = mean difference.

**Table 3:** Side effects of GLP-1 RA and recommendations.

System	Side Effects	Key Notes	Recommendations
Gastrointestinal	Nausea, vomiting, early satiety, bloating, abdominal pain, diarrhea/constipation, delayed gastric emptying	Common at initiation and dose increase, usually transient; may reduce fluid and food intake, promoting dehydration and functional renal failure; diabetic gastroparesis may be aggravated; delayed gastric emptying may alter absorption of other oral medications.	Initiate with a low dose and titrate slowly; administer with light meals; explain symptoms in advance; adjust or temporarily discontinue treatment for severe vomiting or diarrhea; educate to maintain fluid intake; avoid or use with great caution in patients with confirmed gastroparesis; review and, if necessary, shift the schedule for oral medications with a narrow therapeutic window.
Biliar/pancreatic	Acute pancreatitis, gallstones	Pancreatitis: causal relationship incompletely clear; increased risk of lithiasis and cholecystitis, probably mediated by rapid weight loss and altered gallbladder motility.	Do not initiate in patients with a history of pancreatitis; immediately stop GLP-1 RA if pancreatitis is suspected; monitor clinically for biliary symptoms in patients with gallstones.
Oncologic	Medullary thyroid cancer	It is a class contraindication in patients with a personal or family history of medullary thyroid carcinoma or MEN2 syndrome.	Evaluate for thyroid ultrasound, calcitonin, and thyroid hormones.
Ophthalmology	Progression of diabetic retinopathy, NAION	The increased risk was observed especially with semaglutide.	Initial ophthalmological examination in patients with known retinopathy; avoiding sudden and very pronounced decreases in HbA1c.
Cardiovascular	Modest decrease in blood pressure, slight increase in heart rate	May cause dehydration, symptomatic hypotension, and renal hypoperfusion.	Monitoring BP and symptoms of hypotension in the first weeks and after dose changes; adjusting antihypertensive treatment if BP drops too much; encouraging home monitoring (BP and pulse).

HbA1c, glycated hemoglobin; BP, blood pressure; NAION, non-arteritic anterior ischemic optic neuropathy; GLP-1 RA, glucagon-like peptide-1 receptor agonist.

## Data Availability

No new data were created or analyzed in this study. Data sharing is not applicable to this article.

## Supplementary Materials

The following supporting information can be downloaded at https://www.mdpi.com/article/10.3390/medicina62081509/s1.

## Abbreviations

The following abbreviations are used in this manuscript:

ADA	American Diabetes Association
AGE	advanced glycation end-product
ANP	atrial natriuretic peptide
ASCVD	atherosclerotic cardiovascular disease
BP	blood pressure
cAMP	cyclic adenosine monophosphate
CKD	chronic kidney disease
CV	cardiovascular
CVOT	cardiovascular outcome trial
DKD	diabetic kidney disease
DPP-4	dipeptidyl peptidase-4
EASD	European Association for the Study of Diabetes
eGFR	estimated glomerular filtration rate
ESC	European Society of Cardiology
GFR	glomerular filtration rate
GIP	glucose-dependent insulinotropic polypeptide
GLP-1	glucagon-like peptide-1
GLP-1 RA	glucagon-like peptide-1 receptor agonist
HbA1c	glycated haemoglobin
KDIGO	Kidney Disease: Improving Global Outcomes
MACE	major adverse cardiovascular events
NHE3	sodium-hydrogen exchanger 3
PKA	protein kinase A
RAAS	renin–angiotensin–aldosterone system
RCT	randomized controlled trial
SBP	systolic blood pressure
SGLT2i	sodium–glucose cotransporter-2 inhibitor
T2D	type 2 diabetes
UACR	urinary albumin-to-creatinine ratio
