# Supplementary material for: Renal Effects of Glucagon-like Peptide-1 Receptor Agonists in Diabetic Kidney Disease: A Narrative Review of Mechanisms and Clinical Evidence
Source: Medicina (Kaunas). 2026 Aug 5;62(8):1509. doi: 10.3390/medicina62081509 (PMC13514747; doi:10.3390/medicina62081509)
Supplement: Supplementary file 1 [file medicina-62-01509-s001.zip › medicina-4382975-supplementary.pdf]

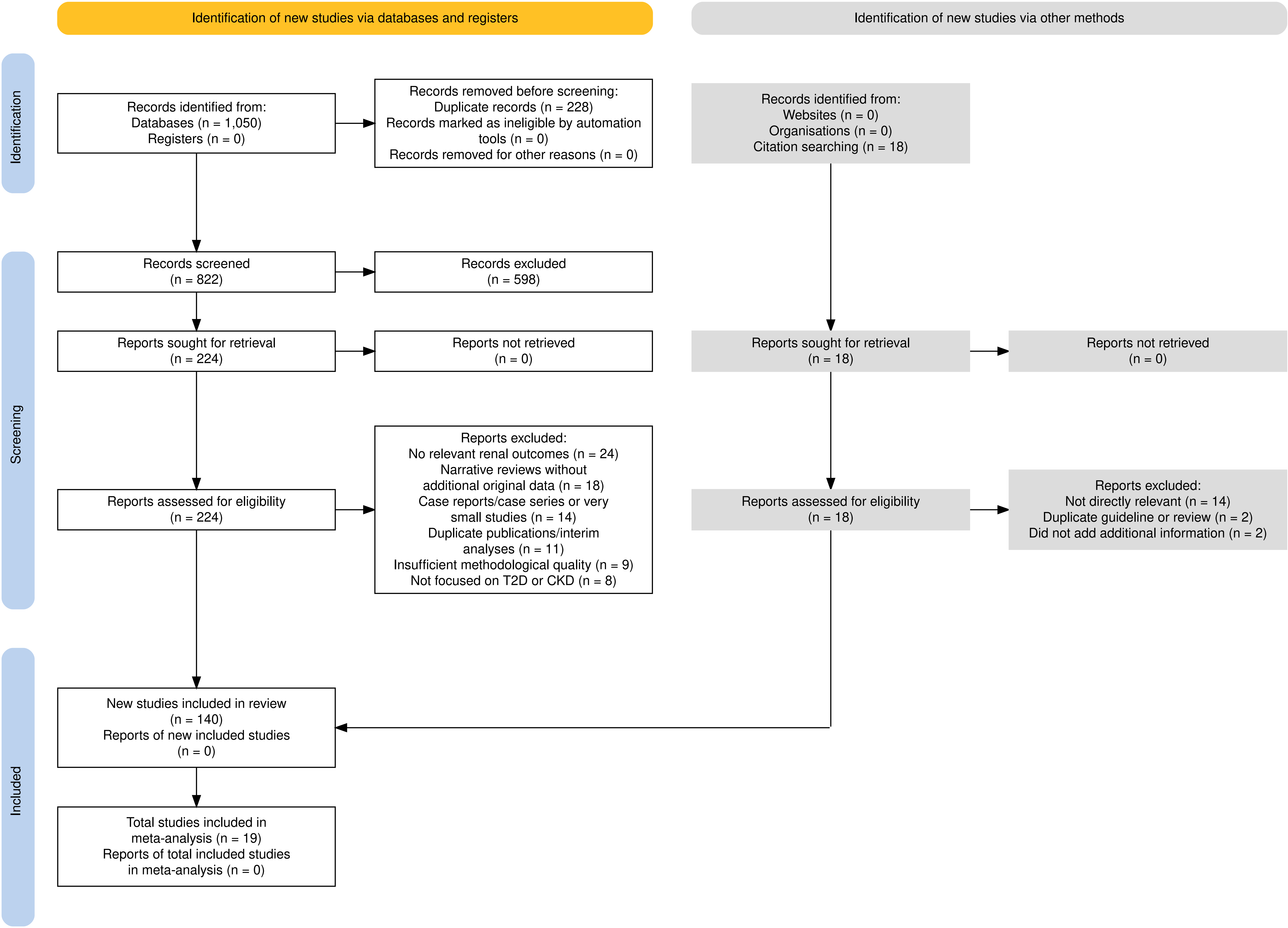
Identification of new studies via databases and registers
Identification of new studies via other methods
Identification
Screening
Included
Records identified from:
Databases (n = 1,050)
Registers (n = 0)
Records removed before screening:
Duplicate records (n = 228)
Records marked as ineligible by automation tools (n = 0)
Records removed for other reasons (n = 0)
Records identified from:
Websites (n = 0)
Organisations (n = 0)
Citation searching (n = 18)
Records screened
(n = 822)
Records excluded
(n = 598)
Reports sought for retrieval
(n = 224)
Reports not retrieved
(n = 0)
Reports sought for retrieval
(n = 18)
Reports not retrieved
(n = 0)
Reports excluded:
No relevant renal outcomes (n = 24)
Narrative reviews without additional original data (n = 18)
Case reports/case series or very small studies (n = 14)
Duplicate publications/interim analyses (n = 11)
Insufficient methodological quality (n = 9)
Not focused on T2D or CKD (n = 8)
Reports assessed for eligibility
(n = 224)
Reports assessed for eligibility
(n = 18)
Reports excluded:
Not directly relevant (n = 14)
Duplicate guideline or review (n = 2)
Did not add additional information (n = 2)
New studies included in review
(n = 140)
Reports of new included studies
(n = 0)
Total studies included in meta-analysis (n = 19)
Reports of total included studies in meta-analysis (n = 0)
